# Improving functional outcomes and quality of life in an elderly woman with sarcopenia and spinal Tuberculosis: A case report

**DOI:** 10.3389/fresc.2023.1076010

**Published:** 2023-02-03

**Authors:** Irma Ruslina Defi, Nur Rusyidah Hamam, Vitriana Biben, Nuzula Chafidh Al Barqi

**Affiliations:** ^1^ Physical Medicine and Rehabilitation Department, Hasan Sadikin General Hospital, Faculty of Medicine Padjadjaran University, Bandung, Indonesia; ^2^ Medical Faculty of Sebelas Maret University, Bandung, Indonesia

**Keywords:** activity of daily living, elderly, physical medicine and rehabilitation, quality of life, spinal cord injury

## Abstract

**Background:**

Indonesia is the world's second-largest contributor to TB patients. According to prevalence by age, the elderly have the most diagnosed TB. In Indonesia, TB spondylitis affects approximately 5% of TB cases and is a common cause of non-traumatic spinal cord injury (NTSCI). Spinal cord injury (SCI) is a growing public health concern, particularly among the elderly, as many of its populations have sarcopenia. Due to the complete absence of voluntary muscle contraction, SCI is followed by a rapid loss of skeletal muscle mass. SCI has several physical, psychological, social, and economic consequences.

**Case presentation:**

A 68-years-old woman presented with weakness and numbness of all four limbs. She also had sarcopenia, malnutrition, and dependency on activities of daily living (ADL). In addition, the patient was at a risk of SCI complications. Magnetic resonance imaging (MRI) showed destruction of the vertebral bodies at the level of the 5th and 6th cervical area, tuberculous abscesses of the paravertebral and longus colli muscles. The patient underwent debridement and spinal stabilization. However, the patient was at a risk of developing SCI complications. In these patients, the Physical Rehabilitation and Medicine (PRM) strategy focuses on improving medical conditions, including preventing secondary complications, promoting neurological recovery, and optimizing function.

**Conclusion:**

This case highlights the importance of PRM intervention in assessing functional disorders in the elderly to improve their quality of life (QOL).

## Introduction

Indonesia leads the list of the largest contributors to Tuberculosis (TB) patients, the first in Southeast Asia and the second in the world (1). The prevalence of TB in Indonesia in 2018 was 0.4%, with 1,017,290 patients (2). The prevalence by age shows that the elderly group has the most diagnosed Pulmonary TB. The comparison of prevalence between men and women diagnosed with pulmonary TB is almost balanced (3). Bone TB accounted for 10% of extrapulmonary TB cases. 50% of bone TB infections are caused by spinal TB (4).

The global prevalence of spinal cord injury (SCI) ranged from 236.0 to 1,298.0 per million people. Despite the regional differences in SCI prevalence rates worldwide, there has been a trend toward increasing prevalence rates in recent decades (5). In 2014, 104 SCI cases were registered at the Fatmawati General Hospital in Indonesia, with 37 being traumatic and 67 being non-traumatic. Car accidents and falls from great heights were the most common causes of traumatic SCI, while infection and neoplasm were the most common causes of nontraumatic-SCI (NTSCI) (6). In Ethiopia, Kenya, and Malawi, tuberculosis was a significant cause of NTSCI (7). SCI is a major public health issue due to its physical, psychological, social, and economic consequences (8).

The prevalence of sarcopenia varies among different populations (9). Even in healthy populations, a recent systematic review found that a significant proportion of the elderly have sarcopenia (10). Sarcopenia is associated with poor health outcomes, decline in quality of life (QOL) and disability risk (11).

Despite the fact that both sarcopenia and SCI are associated with skeletal muscle function and mass loss, SCI is associated with severe atrophy of both Type I and II muscle fibers, with a 90% shift toward Type II fibers (12). In SCI, the total loss of neurological input is followed by a rapid loss of skeletal muscle mass due to the complete absence of voluntary muscle contraction (13).

Physical and Rehabilitation Medicine (PRM) interventions aim to improve physical, cognitive, and emotional disorders while accommodating discharge goals and plans. The treatment includes psychoeducation for patients and families, information about relevant community strategies, and supportive assistance for patients, families, and caregivers. Interventions by the PRM team is appropriate for the clinical presentation of SCI. This multidisciplinary team comprises a physiatrist (PRM doctor), physiotherapist, occupational therapist, speech pathologist, psychologist, and social worker(14). This case report aims to provide insight into the benefit of PRM intervention to improve functional outcomes for an elderly patient with sarcopenia and SCI.

## Case description

A 68-years-old woman presented with weakness and numbness of all four limbs. The patient consented to participate in this case report. She could slide her legs sideways but was unable to lift them. She was able to move both arms with better movement in the left arm. However, she had difficulty clenching and grasping objects using her hands and fingers. The patient was right-handed. She was able to turn over in bed and sat reclining with the caregiver's assistance. All of her daily activities were performed with assistance, and she could not sit without support. She also could not feel the urge to urinate and defecate, and had incontinence.

She felt pain in the middle back, which radiated to her legs, with a numerical rating scale (NRS) of 7. The pain was sharp and was accompanied by a burning sensation unrelated to any activity. The patient was administered gabapentin 300 mg daily for the last two weeks, and the NRS score was reduced to 5. The patient also experienced localized pain in her right shoulder and buttocks, with an NRS of 5, respectively. In addition, she complained of bilateral knee pain with an NRS score of 3. The patient often refused to be seated in a reclined position and preferred to lie prone all day. She slept a lot during the day and had difficulty sleeping at night.

**Figure 1:**
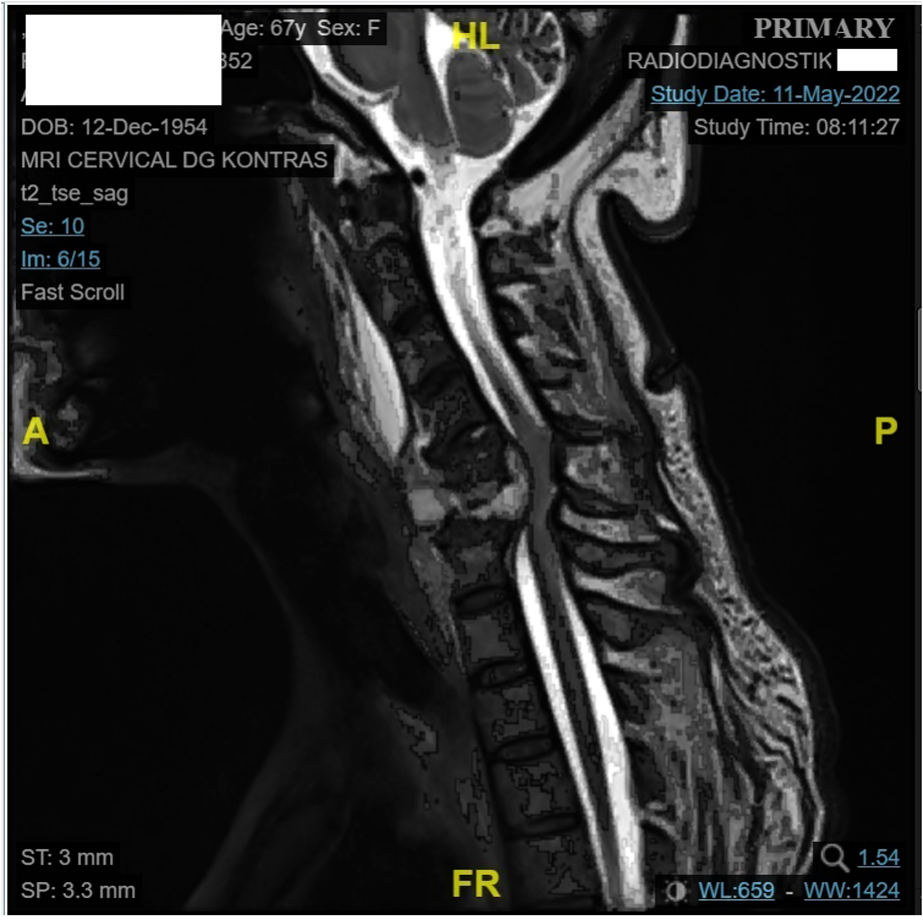
MRI of the patient. Vertebral body destruction of C5 and C6 accompanied by signs of myelopathy, suggestive paravertebral abscess measuring 1.54 x 3.50 x 1.67 cm, infiltrating m. long of neck bilateral, retrolisthesis of the C6 to C7 vertebral bodies posteriorly with a displacement of >50%. Supporting the diagnosis of SCI of our patient.

She also experienced malaise, epigastric discomfort, nausea, episodic vomiting, and a yellowish appearance on the sclera and skin in the previous week. She lost her appetite, which resulted in decreased food and drink intake, although she had no difficulty swallowing liquids or solid foods. The presumptive diagnosis was spondylitis tuberculosis. She had consumed anti-tuberculosis drugs for two months. Magnetic resonance imaging (MRI) revealed destruction of the vertebral bodies at the level of the 5th and 6th cervical area, with paravertebral and longus colli muscle abscesses (Figure 1). Subsequently, she underwent debridement and spinal stabilization surgery. The spine stabilization procedure was anterior cervical corpectomy decompression and fusion (ACDF).

Her body mass index (BMI) was 14.98 kg/m^2^ (categorized as underweight). Her vital signs were uneventful, and communication was good. Both the sclera and skin were icteric. No palpable enlargement of the liver or spleen was observed. She also had a pressure injury at the sacrum measuring 2 × 1 cm, grade II. This patient had a limited range of movement (ROM) in the neck and upper extremities. She also had limited flexion in all parts of the metacarpal joints. Neurological examination revealed that she had SCI with an Asia Impairment Score (AIS) grade C and neurological level (NL) at the 5th cervical area (SCI AIS C NL C5). The patient wishes to return to her role as a grandmother and perform activities in a sitting position.

We found that this patient was categorized as malnourished, with a score of 4, as assessed using the validated Mini Nutritional Assessment (MNA) tools (15). She also had a mid-upper arm circumference of 17 cm, categorized as low circumference (16). She had mild cognitive impairment (MCI) with a score of 22 as measured using Montreal Cognitive Assessment Indonesian version (MOCA-INA) (17). This patient also had suggestive sarcopenia with a score of 18 on the SARC-Calf score (18). We could not perform a bioelectrical impedance analysis (BIA) to confirm the diagnosis of sarcopenia because the patient could not stand properly on her own. We also tried using a hand dynamometer to measure the strength of the hand grip, but the patient was unable to press the handle maximally due to the stiffness of the metacarpophalangeal (MCP) joints in both hands. In addition, she was considered inactive according to the International Physical Activity Questionnaire (IPAQ) (19). She was also regarded as dependent, with a score of 21/100 on Spinal Cord Independence Measurement (SCIM) with poor QOL (20, 21). This patient had probable depression based on the Geriatric Depression Scale (GDS) (22). Her caregiver has a caregiver burden, scoring 44, based on the Zarit Burden Scale (ZBS) (23).

She received pain management therapy during hospitalization. We used extracorporeal shock wave therapy (ESWT) and infrared radiation (IRR) to manage the right shoulder pain due to tendonitis, with an NRS score of five. Both were assigned to the insertion of the rotator cuff muscle area. ESWT was performed once a week, and IRR was performed twice a week. Laser therapy has been used to treat buttock pain caused by piriformis syndrome. It was administered to the piriformis muscle twice a week. All therapy modalities will be administered for one month and will be evaluated monthly.

**Figure 2:**
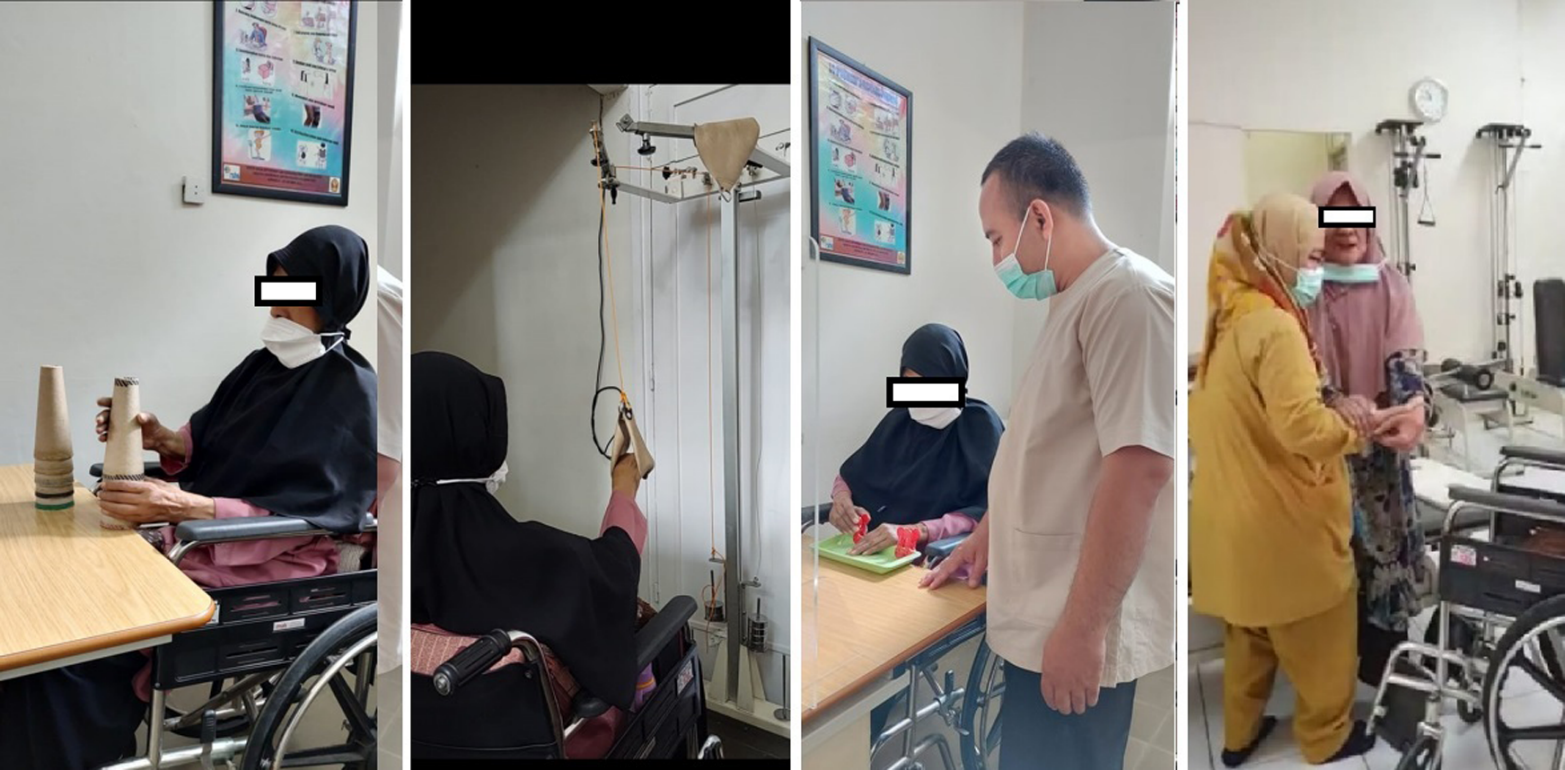
PRM program was given to our patient.

Our patient performed the 3-month outpatient PRM program after the initial assessment with flexibility and strengthening exercises for the extremities (Figure 2), as well as activity of daily living (ADL) adjustment training (for eating, grooming, and upper dressing). Additionally, she received psychological support from a psychologist. The short-term goals of our PRM intervention were increasing body weight, reducing shoulder pain, ensuring appropriate sleep time, independent ADL for eating, grooming, and upper body dressing, partial dependency on bed mobilization, partial dependency on transfer, managing depression, and no SCI complications. The long-term goals for the patient include managing tuberculosis, increasing the QOL, and reversing the sarcopenia condition.

## Discussion

This report describes the case of a 68-year-old right-handed female with tetraplegia due to SCI caused by tuberculosis spondylitis. SCI caused by tuberculosis spondylitis generally has a good prognosis. However, the combination of SCI with older age requires the following: SCI in the elderly precipitates the aging process, which can accelerate the decline in physical health, especially in physical independence, mobility, work function, social integration, and overall QOL. Medical status, including response to anti-tubercular treatment (ATT), geriatric syndromes, and comorbidities, determines patient prognosis (24, 25).

The patient did not receive immediate treatment at the onset of the SCI. Four weeks later, she underwent appropriate management and stabilization surgery. She had incomplete motor and sensory impairments starting at the level of the 5th cervical area, with moderate-to-severe weakness in the upper extremities, trunk, and lower extremities. Therefore, the patient was completely dependent on performing all activities of daily living (ADL), transfer, and mobility, leading to prolonged immobilization.

She had muscle atrophy and pressure sores due to physical inactivity and was immobilized. Malnutrition, sarcopenia, mild cognitive impairment, and depression, in addition to caregiver burnout, can slow rehabilitation programs. Moreover, ATT was postponed due to anti-tuberculosis drug-induced liver injury (AT-DILI) (25, 26).

A different approach is required to treat this patient. Malnutrition, being female, and older age increased the risk of AT-DILI in these patients (27, 28). In our patient, it was necessary to ensure adherence to ATT treatment, adequate nutritional intake, and good wound care. After the internal medicine specialist discontinued and reintroduced the ATT regimen and adjusted nutrition management for six weeks, the patient’s medical condition was relatively stable, she also improved her urge to urinate and defecate.

The Guideline Development Group (GDG) unanimously agreed that the feasibility of early rehabilitation is likely to vary depending on patient characteristics and healthcare systems (29). Management of the subacute phase of treatment focuses on improving medical conditions, including preventing secondary complications, promoting neurologic recovery, and maximizing function. Due to the multiple risks of SCI-associated complications caused by cervical lesions, the priority is to resolve the medical problems, followed by rehabilitation programs with a multidisciplinary approach involving a physiatrist for assessment and intervention, a psychologist, a physical therapist, and an occupational therapist (30–32).

Both sarcopenia and SCI cause significant changes in type II muscle fibers (12, 33). Type II muscle fiber atrophy is widespread in elderly female sarcopenic patients (33). A shift toward more Type II muscle fibers is characteristic of SCI (12). Kosek et al. said prolonged strengthening type exercise training can reverse much of the decline in Type II muscle fiber size in the elderly (34). We prescribed strengthening exercises for the extremities. The focus of our PRM mobilization program, were the flexibility and strengthening exercises for the extremities, and ADL adjustment training (Table 1).

**Table 1:** Timeline of our patient episode care.

	1 Month after initial visit		2 Month after initial visit		3 Month after initial visit	
Subjective	Malaise reduced, Her eating quantity increased. She was eating independently. Pain in shoulder (NRS 3), buttock (NRS 3), and knee (NRS 3). The patient can tolerate sitting upright position for 15 min.	The patient could maintain sitting at the edge of the bed and wear a mask and instant veil. Her appetite has increased. Pain in shoulder (NRS 3), buttock (NRS 3), and knee (NRS 3).	The patient increased sitting tolerance and could do standing with support. Could wear mask and instant veil. her appetite has increased. Pain in shoulder (NRS 1), buttock (NRS 1), and knee (NRS 2).
Objective	• Body weight • Body height • BMI • Sclera icteric • Extremity • Integument • Spasticity • Muscle spasm • SCIM • GDS • Moca-INA • Zarit burden	: 36 kg : 155 cm : 15 : - : Limited ROM in shoulder, wrist and finger : skin ulcer (−) : knee ekstensor bilateral (MAS 1) : Rotator cuff muscle and piriformis muscle : 27/100 : 5 : 24 : 30	• Body weight • Body height • BMI • Sclera icteric • Extremity • Integument • Spasticity • SCIM • GDS • Moca-INA • Zarit burden	: 39.5 kg : 155 cm : 16.49 : - : Limited ROM in shoulder, wrist and finger : skin ulcer (−) : knee ekstensor bilateral (MAS 1) : 27/100 : 4 : 22 : 24	• Body weight • Body height • BMI • Sclera icteric • Extremity • Integument • Spasticity • SCIM • GDS • Moca-INA • Zarit burden	: 39.5 kg : 155 cm : 16.49 : - : Limited ROM in shoulder, wrist, and finger : skin ulcer (−) : knee ekstensor bilateral (MAS 1) : 35/100 : 4 : 21 : 20
Assesment	• Musculoskeletal pain • Psychological, ADL, and mobilization disturbance • SCI AIS C NL C5 post ACDF 2 months due to spondylithis tuberculosis • ATT-DILI • Osteoarthritis of knee	• Musculoskeletal pain • Psychological, ADL, and mobilization disturbance • SCI AIS C NL C5 post ACDF 3 months due to spondylithis tuberculosis • ATT-DILI on ATT reintroduce • Osteoarthritis of knee	• Musculoskeletal pain • Psychological, ADL, and mobilization disturbance • SCI AIS C NL C5 post-ACDF 4.5 months due to spondylithis tuberculosis • Osteoarthritis of knee
Plan	• ATT reintroduce • Nutritional management program • Wound care management and laser theraphy • Psychological counselling for patient and family • ESWT and IRR modality for shoulder • Laser therapy for buttock • Bed mobility, sitting upright tolerance training • Transfer training for caregiver • ADL training in sitting position • Home exercise program	• ATT reintroduce • Nutritional management program • Psychological counselling for patient and family • ESWT and IRR modality for shoulder • Laser therapy for buttock • Supported standing training • Transfer training with pivot • Continued ADL training in sitting position • Home exercise program	• ATT reintroduce • Nutritional management program • Respiratory muscle endurance training using incentive spirometry • Walking training using walker • Ocupational Therapy for Upper Extremity strenghtening and task spesific training program • Home exercise program

NRS, numeric rating scale; BMI, body mass indeks; ROM, range of motion; MAS, modified ashworth scale; SCIM, the spinal cord independence measure; GDS, geriatric depression scale; Moca-INA, montreal cognitive assesment indonesian version; ADL, activity of daily living; ACDF, anterior cervical corpectomy decompression and fusion; ATT, anti-tuberculosis treatment; DILI, drug-induced liver injury; ESWT, extracorporeal shock wave therapy; IRR, infrared radiation.

It is also important to prevent conditions that can further reduce the functional capacity. We treated her shoulder pain due to tendonitis and buttock pain due to piriformis syndrome. ESWT was effective and safe for the treatment of tendonitis (35). Shockwaves alleviate tendon pain through hyper-stimulation analgesia and reduced substance P release from the treated region (36). In managing musculoskeletal pain, infrared radiation causes an intracellular increase in reactive oxygen species (ROS), followed by an increase in nitric oxide (NO) synthesis and intracellular calcium levels (Ca2+). These reduce oxidative stress, induce vasodilation, and stimulate growth factor production and extracellular matrix deposition, ultimately leading to tissue repair (37). The laser induces an analgesic effect in piriformis syndrome by positively impacting chondrocyte proliferation and matrix synthesis. Laser therapy has anti-inflammatory and anti-edematous effects owing to its influence on prostaglandin synthesis (38).

Increasing patient independence will positively reduce the caregiver burden and provide good social support. Support can be obtained with immediate family cooperation in caring for patients with SCI (39). We provided counselling support by a psychologist for patients and families to overcome obstacles and engage them to formulate rehabilitation goals together.

Scivoletto reported that more than four points of change in the SCIM score showed clinically significant improvement (40). During follow-up, our patient's SCIM scores increased from 23 to 35 after three months of outpatient rehabilitation programs, which reflected an improvement in functional ability. She also showed improved motor strength in the upper and lower extremities. She was able to maintain a sitting balance and perform ADLs in a sitting position, eating, drinking, grooming independently, and upper body dressing with moderate assistance. She could move from lying down to sitting on the edge of the bed with minimal assistance, standing and pivoting to a wheelchair with maximum assistance. Although our patient was still unable to maintain a standing position, she succeeded in reaching the short-term goals of our PMR intervention.

From the last follow-up, our patient felt a lack of confidence due to the pain and fear of falling. This condition can be overcome by providing exercises to improve balance and coordination during static standing. The MCI we found in our patient made her learn new skills longer than usual. However, challenging programs that provide new activities, learning, and exercise can trigger the brain to grow and recover from brain senescence and help improve MCI. Closed monitoring is still needed to adjust patient management and monitor disease progression to further improve functional abilities.

Although SCI caused by tuberculosis spondylitis has a good prognosis, we have added evidence by describing another case of SCI rehabilitation management. No complications or adverse events were reported from the PRM program point of view. Our patient also showed successful improvement in ADL ability and QOL. However, our study had several limitations. First, there was a single clinical case report with fewer factors for analysis and a lack of quantitative data strength and consistency in terms of evidence. The clinical assessment of muscular spasms has only prior initial parameters and no final parameters, partly because the assessment instruments make the evaluation in pre- and post-moments large in time. The muscular spasm was found at the area of the piriformis muscle and rotator cuff muscle. Another limitation of this case report is the absence of a urodynamic examination. Patient presented urinary incontinence although sensory of S4-S5 (pin-prick & light touch), DAP (deep anal pressure) and VAC (voluntary anal contraction) were all tested normal. Urodynamics were suggested at the follow-up to assess detrusor overactivity, dyssynergia and compliance. Patient refused urodynamics and close follow-up for neurogenic bladder and bowel dysfunction (i.e., incontinence, urinary tract infection).

## Patient perspective

“After I underwent a rehabilitation program, I continue improving,” the patient exclaimed gracefully. “I improved my leg strength to the point where I could stand on it, which I had never done before since I was sick. It changed my life, even though I still needed an assistive device to help me stand.”

## Conclusion

This case highlights the importance of a rehabilitative intervention to detect functional disorders in the elderly, as it can help patients with SCI to improve ADL's ability and QOL.

## Data Availability

The original contributions presented in the study are included in the article, further inquiries can be directed to the corresponding author/s.
